# Neurophysiology goes wild: from exploring sensory coding in sound proof rooms to natural environments

**DOI:** 10.1007/s00359-021-01482-6

**Published:** 2021-04-09

**Authors:** Heiner Römer

**Affiliations:** Department of Biology, Graz University, Universitätsplatz 2, 8010 Graz, Austria

**Keywords:** Sensory coding, Transmission channel, Masking, Acoustic communication, Insects

## Abstract

To perform adaptive behaviours, animals have to establish a representation of the physical “outside” world. How these representations are created by sensory systems is a central issue in sensory physiology. This review addresses the history of experimental approaches toward ideas about sensory coding, using the relatively simple auditory system of acoustic insects. I will discuss the empirical evidence in support of Barlow’s “efficient coding hypothesis”, which argues that the coding properties of neurons undergo specific adaptations that allow insects to detect biologically important acoustic stimuli. This hypothesis opposes the view that the sensory systems of receivers are biased as a result of their phylogeny, which finally determine whether a sound stimulus elicits a behavioural response. Acoustic signals are often transmitted over considerable distances in complex physical environments with high noise levels, resulting in degradation of the temporal pattern of stimuli, unpredictable attenuation, reduced signal-to-noise levels, and degradation of cues used for sound localisation. Thus, a more naturalistic view of sensory coding must be taken, since the signals as broadcast by signallers are rarely equivalent to the effective stimuli encoded by the sensory system of receivers. The consequences of the environmental conditions for sensory coding are discussed.

## Introduction

To display adaptive behaviour, animals must collect information about the “outside” physical world using their sensory systems and brains. In humans and animals with nervous system alike, sensory information is transmitted via afferent nerves and encoded in trains of action potentials. The brain, as it decodes this information, has to make assumptions about what has happened in the physical world. A central issue in sensory physiology, therefore, deals with the coding and decoding mechanism(s) in the sense organs and CNS, respectively.

What strategies do sensory systems use to faithfully represent the complex physical world in the simple trains of all-or-nothing action potentials in afferent sensory neurons? Sixty years ago, Barlow first formulated the “efficient coding hypothesis” (Barlow 1961), suggesting that the statistical structure of natural stimuli is already important in the sensory periphery, as it enables the organism to represent the sensory world as a series of discrete APs. In the meantime, enough empirical evidence that supports Barlow’s hypothesis has been accumulated. This evidence shows that sensory systems provide the CNS with less information about artificial stimuli than about behaviourally relevant stimuli (e.g. Rieke et al. 1995; Machens et al. 2001, 2003; Lewicki 2002; Edin et al. 2004). However, as stated by Machens et al. (2005), the process of testing Barlow’s hypothesis is not at all trivial, because it depends on the examined distribution of natural stimuli. These stimulus distributions may differ in many ways with respect to the behavioural relevance, to both inter- and intraspecific variation, or to environmental variation. Thus, researchers must apply particular strategies to search for natural stimuli that are both behaviourally the most relevant and provide the nervous system with the greatest amount of information. Suga used the term “information bearing elements” for those stimulus parameters or parameter combinations that are most relevant for processing biologically important sounds (Suga et al. 1978; Suga 1989).

My review takes a historical perspective to demonstrate how the search for and analysis of these relevant stimuli has changed over time. I chose the auditory system of insects as subject matter for very good reasons: Grasshoppers, crickets, katydids, and cicadas are well-known for their intraspecific acoustic communication, which is critical for the reproductive success of signallers and receivers (Gerhardt and Huber 2002). The repertoire of signals used for communication in each species is small, and a statistical analysis of variation in the signal properties may reveal their potential for encoding biologically important information. Moreover, phonotaxis in crickets and katydids could reliably be elicited, first in arena trials and later with additional, sophisticated walking compensators, Kramer treadmills (Kramer 1976), or trackball systems to monitor subtle details of the receivers’ movements towards (or away) from a sound source (Wendler et al. 1980; Weber et al. 1981; Hedwig and Poulet 2005). In a similar way, the reliable responses of male and female grasshoppers in their duetting communication allowed behavioural approaches to be taken and combined with variations of the species-specific song models to study frequency, loudness, or temporal patterns and to draw conclusions about the sensory systems underlying species recognition and mate choice (von Helversen and von Helversen 1994, 1997; Gerhardt and Huber 2002). The nervous system of animals could be treated as a “black box”, and it could be assumed that natural or sexual selection has provided individuals with the necessary neuronal machinery to perform a given task sufficiently well. Some researchers of animal behaviour, therefore, have questioned the role that neurophysiological approaches could play for explaining behaviour. Earlier in my own career, I was called a “*Neuronenstecher*” (someone who jabs nerve cells with sharp electrodes) by a renowned professor in the field of animal behaviour, to express his doubts about a physiologist’s ability to make substantial contributions to the field of behavioural studies.

However, over the subsequent years, scientists have pointed out the benefits of considering the sensory and cognitive mechanisms that underlie important behavioural decisions (Guilford and Dawkins 1991; Endler 1993; Chittka 1997; Bateson and Healy 2005; Miller and Bee 2012). Indeed, a major advantage of most insects’ auditory systems is that all of the biologically relevant information in an acoustic signal is usually encoded in the activity of a few afferents and is conveyed to the brain by a handful of interneurons. These systems allow physiologists to easily access and study the activity of single, identified afferents or interneurons in response to the very same stimuli that have turned out to be important in behaviour (Gerhardt and Huber 2002). Moreover, hearing in insects also or even primarily evolved for predator detection (Hoy 1992; Fullard 1998; Conner and Corcoran 2012; Yager 2012; Pollack 2015), so that sensory coding can be further studied in a rather different context for stimuli that provide the most obvious fitness consequences.

I start my review with a description of how early researchers studied auditory systems using simple, artificial acoustic stimuli to characterize the range and limits of hearing. Virtually all studies were performed under laboratory conditions, with a seminal exception of Roeder´s outdoor attempt for the coding of bat sound by moth auditory receptors (Roeder and Treat 1957). Technical advances in recording and staining techniques later allowed a comparison between homologous neurons of different species, demonstrating that not all coding properties are adaptations to species-specific signals. Rather, they may represent receiver biases as a result of selection unrelated to the coding of species-specific signals. The natural environment as a transmission channel for sound, with all its abiotic properties and background noise largely determines the sound signal available for a receiver. I discuss an approach for recording single-cell activity with a portable device in the field, and how it can be used for a more naturalistic view of sensory coding of signal patterns and directionality. Finally, these outdoor conditions are also relevant for the other main task of hearing in insects, namely predator detection. I present a case study between rainforest crickets and predatory bats showing how a simple decision criterion may help to separate irrelevant background noise from dangerous, nearby bats.

## Early exploration of auditory systems used simple, artificial stimuli

To study proximate aspects of hearing, simple artificial stimuli are quite appropriate. The basic physiology of sound reception in Orthoptera was first described by Pumphrey and Rawdon-Smith (1936a, b), Pumphrey (1940), and Autrum (1941). As these studies were interrupted by the World War II, some time elapsed before Haskell (1956) claimed that these earlier studies had used rather artificial acoustic stimuli which differed from the normal stridulatory signals of grasshoppers. Still, due to the difficulties faced when attempting to reproduce natural insect songs as stimuli at the time, Haskell still used artificial, pure-tone sound pulses, but varied the pulse repetition rate to demonstrate that the tympanal organs of the four investigated grasshopper species fired volleys of APs up to repetition rates of 90–100 pulses/s.

Physiologists soon began to explore the capacity of insects to discriminate between carrier frequencies of sound. Although the locust rarely displayed any interesting acoustic behaviour as compared to the elaborate communication behaviour of the smaller grasshoppers, it was chosen because it was a larger model and access to both the ear, located in the first abdominal segment, and to interneurons of the auditory pathway was easier. Based on physiological recordings of (unidentified) neurons in the CNS of locusts, Horridge (1960) concluded that they are able to perform some kind of frequency discrimination. This finding was later confirmed by other researchers, who demonstrated that different groups of sensory cells in Müller’s organ in the locust’s ear have attachment points at different locations on the tympanal membrane and are tuned to different frequencies (Michelsen 1968, 1971; Römer 1976; Miller 1977; Jacobs et al. 1999).

Even more elaborate frequency discrimination was found in the ears of katydids, where single receptors in the linear array of the so-called crista acustica are tuned to different sound frequencies. A systematic relationship was identified between the position of the receptor within the ear and the frequency to which it is the most sensitive (Oldfield 1982; Stumpner 1996; Stölting and Stumpner 1998). The tonotopic organisation established in the periphery is maintained in the auditory neuropil of the prothoracic ganglion, as shown by the spatial distribution of the endings of the receptor axons located there (Oldfied 1983; Römer 1983; Stumpner 1996; Stölting and Stumpner 1998). Traveling waves were later established as the mechanical basis for frequency discrimination in locust and katydid ears, using modern techniques like Laser Doppler Vibrometry and scanning LDV (Windmill et al. 2005; Palghat Udayashankar et al. 2012; Montealegre et al. 2012), revealing functional analogies to the mechanism of frequency discrimination in the mammalian ear.

The auditory pathway in the locust’s CNS was first explored with extracellular recordings that used artificial sine stimuli to identify “types” of interneurons solely based on their tonic, phasic, or phasic-tonic response patterns to pure-tone stimuli and the tuning of their responses (e.g. Kalmring 1971). These recordings were documented with a camera (Recordine) positioned in front of the oscilloscope screen. Three to five responses were photographed, and one response that was considered as “typical” for the neuron type was later presented in a publication. In this way, the variability among the responses was completely ignored, representing an important constraint for the reliable processing by the CNS, underlying recognition and classification of acoustic stimuli. A considerable amount of time elapsed before the various sources of spike train variability and their different implications with respect to the detection, recognition, and classification task in the auditory system were investigated (Ronacher and Römer 1985; Machens et al. 2003; Vogel et al. 2005; Ronacher 2014; review in Ronacher et al. 2004).

Having access to a soundproof room in the sixties and seventies of the last century was (and still is) a great advantage, particularly for studying the biophysics of directional hearing, since such experiments require the use of an experimental set-up which guarantees that the animal’s ears receive sound only from the intended direction. When taking electrophysiological approaches to study the auditory system under laboratory conditions, it is often necessary to invest a great deal of effort to reduce or eliminate the potential scattering effects of micromanipulators, animal holders, or other equipment. Some reviewers gave researchers a hard time to get their manuscripts on insect hearing published, if there was only little doubt about the acoustic conditions in their experiments. In dichotic stimulation experiments using earphones for crickets and katydids (Kleindienst et al. 1981; Rheinlaender et al. 2006) or piezo-electrical transducers in locusts (Rheinlaender and Mörchen 1979) the acoustic conditions in the free field either played no role or were chosen in such a way that each ear perceived sound only from one side (grasshoppers; Rheinlaender and von Helversen 1988).

Roeder’s attempt to study the coding of predatory stimuli outdoors was in stark contrast to these laboratory-based studies. About the same time as Haskell was studying the responses of grasshopper auditory afferents, Treat (1955) and Belton (1956) published an account on the behavioural responses of moths to ultrasound. A stimulus that could be used effectively to initiate flight escape manoeuvres in moths was the sound produced by a dog whistle or by shaking a bunch of keys. The first neurophysiological responses of the moth’s tympanal organ were reported by Roeder and Treat (1957), who demonstrated the coding of an ultrasonic signal in the AP activity of two receptors in the moth’s ear. But Kenneth Roeder was aware of the fact that the system could not be analysed without interfering with its normal operation. This problem concerned the degree of restriction accompanied by the surgical procedures that were necessary to gain access to the nervous system and, specifically, those needed to either record the AP pattern with hook electrodes from the tympanal nerve or—with even greater interference—with microelectrodes from thoracic ganglia or the brain. He recognised that another limitation of the method was the fact that the whole system had to be placed under controlled conditions so that external variables could be manipulated independently. To maintain the excitability of his preparations but avoid altering the testing process itself, Roeder repeated the acoustic stimuli only once per second. He knew, however, that this was far below the repetition frequency of the cries of a bat to which a moth would be exposed in nature. As Roeder stated “These are never the conditions of normal operation under which the system became adapted to promote survival of the species” (Roeder 1970). And with respect to the escape behaviour of the moth he noted that “No formula or circumstance could be found that would bring performance levels in the laboratory up to those observed in the field”. It was probably the awareness of such difficulties which led him and co-workers to study the operation of the simple auditory system of moths directly in nature. Instead of using artificial bat stimuli, the cries of real bats passing by the preparation at different distances were taken as the most naturalistic stimuli (Roeder and Treat 1957; 1961). In these early days of physiological research on the auditory system in insects, this was quite a modern systems approach. Surprisingly, it took about 30 more years before some of Roeder’s ideas were applied to study the coding of naturalistic stimuli in Orthoptera (see below).

## The identified neuron approach and evolutionary thinking in physiology

Although the monograph “Nerve Cells and Insect Behavior” was published by Roeder (1963) before the general use of single-cell staining techniques, he used the term “neuronal parsimony” to express his belief that insects can perform adaptive behavioural responses with a relatively small number of sometimes large nerve cells. An identified nerve cell is one which can be found in each individual of a species (Kandel 1976). An extracellular recording and staining technique with cobalt was used in the first step toward the identification of neurons in the auditory pathway of locusts (Rehbein et al. 1974; Rehbein 1976), namely, a modification of the intracellular staining method described by Pitman et al. (1972). The search for neuronal elements in the locust’s auditory pathway became a “truly” identified neuron approach as intracellular recording and staining techniques were applied (Römer and Marquardt 1984). Putatively homologous interneurons in different cricket species had been described earlier (Casaday and Hoy 1977; Wohlers und Huber 1978; Popov et al. 1978). In subsequent years, information about identified, apparently homologous nerve cells in related insect species accumulated (Zhantiev and Korsunovskaya 1983; Römer et al. 1988; Stumpner and Molina 2006), so that it became possible to carry out comparative studies and develop evolutionary models to reconstruct neural circuitry (see Comer and Robertson 2001 for a review on identified nerve cells in insects).

Another major step forward in our understanding of the insect auditory system was a series of developmental studies which demonstrated that all central neurons are derived from precursor cells (neuroblasts; Bate 1976) (for an overview of the development of the auditory system, see Boyan 1992). Each ganglion comprises 61 neuroblasts (30 in each hemiganglion and one unpaired), which are organised in a stereotypical way in the grasshopper and all other insects studied. Each neuroblast gives rise to a stereotypic set of progeny; for example, interneuron 714 (formerly named “G-neuron” in studies without morphological identification) could be traced back to neuroblast 7–4 as serially homologous interneurons in all neuromeres between the second abdominal ganglion and suboesophageal ganglia (Boyan 1993). The recognition of such a serially repetitive *Bauplan*, with a basic neuronal organisation reiterated in different segments, allowed to define the extent to which homology results in common neuronal properties (Prier and Boyan 2000). Furthermore, comparative developmental studies demonstrated that the ear of the locust in the first abdominal segment is homologous to the proprioceptive pleural chordotonal organs found in the six other abdominal segments (Meier and Reichert 1990). These findings suggested that insect tympanal organs have evolved from proprioceptors and that the transition between proprioception and exteroception involves minimal neural changes (Fullard and Yack 1993). Indeed, in a primitive atympanate grasshopper, the chordotonal organs arrayed along the abdominal segments of the body wall are all sound-sensitive, respond to sound frequencies and intensities that are biologically significant, and mediate adaptive behavioural responses (van Staaden and Römer 1998). This transition from proprioceptive to exteroceptive function along the array of pleural chordotonal organs provided evolutionary evidence in line with the serial homology demonstrated ontogenetically by Meier and Reichert (1990).

What are the consequences of the conserved basic *Bauplan* of the auditory system for the coding of natural, species-specific stimuli? Given that grasshopper species have a highly accurate ability to distinguish their song from those of other species (von Helversen and von Helversen 1994), their auditory system must be able to solve this task. In addition to identification of conspecific songs, females also discriminate between signal variants of different conspecific males (e.g. Kriegbaum 1989). The task of encoding these signal variants is much more demanding, since the discrimination of basically similar afferent spike trains must be possible, despite their considerable intrinsic variability. Still, Machens et al. (2003) determined that enough information to distinguish these song variants is available in the spike trains of single auditory afferents, if this activity is analysed on an appropriate (ms) time scale.

At the level of thoracic auditory interneurons, Ronacher and Stumpner (1988) described the responses of an interneuron (AN4) in the grasshopper *Ch. biguttulus,* which has filtering properties to small gaps in the syllables of the song. The response of this neuron drops to almost zero when model songs include gaps with a width of 2.5–4 ms. This is the result of the temporal interaction of a short latency inhibitory potential followed by excitatory synaptic potentials. In behaviour, females strongly reject model songs with gaps of the same width. Therefore, it was tempting to speculate that the response characteristics of this neuron regarding temporal parameters, as well as the accurate coding of signal variants in auditory receptors, are species-specific adaptations in *Ch. biguttulus*. However, as discussed by Ronacher and Stumpner, this is not the case. A likely homologue of AN4 was described earlier in *Locusta migratoria* (Römer and Marquart 1984) with an identical temporal interaction of inhibitory and excitatory synaptic potentials. The authors, therefore, suggested that AN4 might be common in most grasshoppers and that it has evolved its physiological characteristics in another, unknown context. Once present in ancient grasshoppers, this neuron served as a preadaptation for gap detection in the mate choice behaviour of *Ch. biguttulus* females. Neuhofer et al. (2008) later went one step further and investigated the evolutionary constraints for sensory coding. They compared the coding properties of many identified, putatively homologous auditory neurons in the locust and *Ch. biguttulus*, two species with an evolutionary history of long separation (Flook and Rowell 1997). The authors argued that they had taken this comparative approach because sound signals and an elaborate duetting communication play important roles in *Ch. biguttulus* but not in the locust. Although they used the most rigorous method available to measure and quantify the similarity of spike trains, the authors detected no significant differences in the responses from interneurons between both species, indicating that their coding properties are an apomorphic, evolutionarily conserved feature (see also Ronacher 2014).

Whereas Neuhofer et al. (2008) compared two species with an evolutionary history of long separation, Kostarakos and Römer (2015, 2018) investigated the coding of conspecific signals in two closely related sibling katydid species in the *Mecopoda elongata* complex. They live and communicate in sympatry with rather different signals. The “chirper” species produces short chirps at a rate of about 0.5/s, and the “triller” produces highly redundant, long-lasting signals at SPLs of more than 100 dB (Siegert et al. 2013). Surprisingly, males of the “chirper” could detect a conspecific chirp in the continuous call of the “triller” at SNRs of − 8 dB, although the spectra of both signals are broadly similar, apart from more energy at 2 kHz in the “chirper” signal. Kostarakos and Römer (2015) described two coding mechanisms in auditory interneurons that result in selective coding of the chirper signal despite the continuous background noise of the “triller” species: “novelty detection” and “selective tuning”. However, the same mechanisms were also found in interneurons of the “triller”. Consequently, these neurons in the “triller” respond only to the “wrong” signal: the heterospecific “chirper” song. Low-frequency tuning and novelty detection do not result from the selection pressure of the sympatric “triller” species. Schul and Sheridan (2006) and Schul et al. (2012) had described the highly selective encoding of bat-like calls in another katydid (*Neoconocephalus retusus*), despite the simultaneous presence of a repetitive conspecific signal. Thus, a ‘novelty detector’ appears to be present in other katydids as well, and this seems to be adaptive in another behavioural context of predator detection. Kostarakos and Römer (2018) suggested that chirpers evolved an additional, 2-kHz component in their song and exploited pre-existing neuronal properties that enable them to detect their song under masking noise. In fact, one important element of the sensory drive model (Endler 1992; Cummings and Endler 2018) is that environmental conditions that are present during signal transmission favour the evolution of signal traits that exploit sensory biases in receivers.

The “pre-existing receiver bias model” suggests that biases can be established in the nervous system of receivers for the signaller trait in a context other than the sexual selection (Endler and Basolo 1998; Ryan and Cummings 2013). Such a bias could exist at any level of a sensory system, from peripheral receptors up to neuronal circuits, and affect the final decision-making. Note that, in the case of such a bias, the naturalistic signal does not yet exist in the sense of the “efficient coding hypothesis”, but the sensory system can be exploited by signallers if they shift their signal into the range of the receiver bias. Two other striking cases of a sensory bias in the auditory system of moths and crickets can be found in Nakano et al. (2008, 2010) and ter Hofstede et al. (2015), respectively.

To summarize, coding properties of neurons may be highly adaptive for filtering out conspecific signals, but even a perfect match between a sensory coding property and a signal feature does not provide conclusive evidence that this property evolved as a specific adaptation to this feature (Chittka and Briscoe 2001). Thus, neurophysiological studies and neural network models (Phelps 2007) have strongly contributed to our understanding of hidden preferences in receivers and the preferences they express in behavioural trials, although the preferences for acoustic signals can be examined without any knowledge of the underlying sensory system. Similarly, the different behavioural paradigms applied in studies of grasshoppers, crickets, and katydids have shown that the innate releasing mechanism for species recognition is based primarily on the temporal pattern of songs (von Helversen 1972; Stumpner and von Helversen 2001; Gerhardt and Huber 2002; Hennig et al. 2004; Ronacher 2019). The search for the underlying neuronal network of the innate releasing mechanism in crickets has been long and difficult. During this time, a shift in the different concepts has been observed (reviewed in Ronacher 2019). Still, the picture that is currently emerging (Kostarakos and Hedwig 2015; Schöneich et al. 2015; Hedwig 2016) classically illustrates the fruitful interaction between behaviour and physiology in the field of neuroethology.

## Increased awareness for properties of the environment as the transmission channel for sound

In this review, I distinguish between “natural” and “naturalistic” stimuli for the following reason. Let us consider the famous experiment performed by Regen more than 100 years ago (Regen 1913). A male cricket was singing in one room, and his calling song (the natural stimulus) was being transmitted via telephone into a neighbouring room, where a female performed phonotaxis towards the telephone speaker. The quality of the sound signal at the receiver’s end (the naturalistic stimulus after transmission) must have been horrible, with distortions in the frequency and time domain, in addition to the cracking noise that was typical for telephone transmission at that time. Nevertheless, the auditory system of the female was able to process this stimulus well enough for her to reach a decision to approach the supposed mate. Whereas the transmission channel for sound was a technical one in Regens’ experiment, the natural environment of signallers and receivers constitutes the transmission channel. The communication system evolved in this natural environment.

The tasks of signal detection, identification, and discrimination–and localisation for most situations—are all aggravated by the sound transmission channel. Morton (1975) and Wiley and Richards (1978, 1982) presented their empirical work on birdsong, showing that the physical properties of different environments (e.g. open grassland and temperate forests) affected the transmission of birdsong in different ways with respect to frequency filtering and distortions in the time domain. They also noted that the signal properties of different species appeared to be adapted to the properties of the transmission channel. As a consequence, the signals from individuals of the same bird species that inhabited very different environments also differed substantially in these environments (Hunter and Krebs 1979). The acoustic adaptation hypothesis (Morton 1975) suggests that the signal design evolved to reduce the detrimental effects of the specific habitats in which a species communicates. Michelsen’s theoretical treatment on sound transmission in different environments (Michelsen 1978; Michelsen and Larsen 1983; see also Forrest 1994) clearly indicated that the sound signals of insects are probably even more strongly affected by habitat effects than bird song due to their higher sonic or even ultrasonic song frequencies.

Thus, we would expect that evolution has matched the design and function of the auditory system to the demands of the ecology of a given species and, ultimately, that neural coding properties of the system can only properly be investigated in the context of its natural environment. This finally prompted the approach of bringing a preparation with recordings of activity of auditory neurons into the wild, adopted from the original one of Roeder and Treat (1957) with the moth preparation. Taking this approach allowed to study sensory coding where hearing evolved (Rheinlaender and Römer 1986; for further studies, see below). The transmission channel, with all its biotic and abiotic properties, is part of the “sensory drive model” (Endler 1992; Cummings and Endler 2018), which also includes receiver and signal properties as characters which can be predicted based on features in the environment. The sensory coding of naturalistic stimuli is part of this framework. Understanding more about this coding may help us to focus on evolutionary aspects of hearing.

**Fig. 1:**
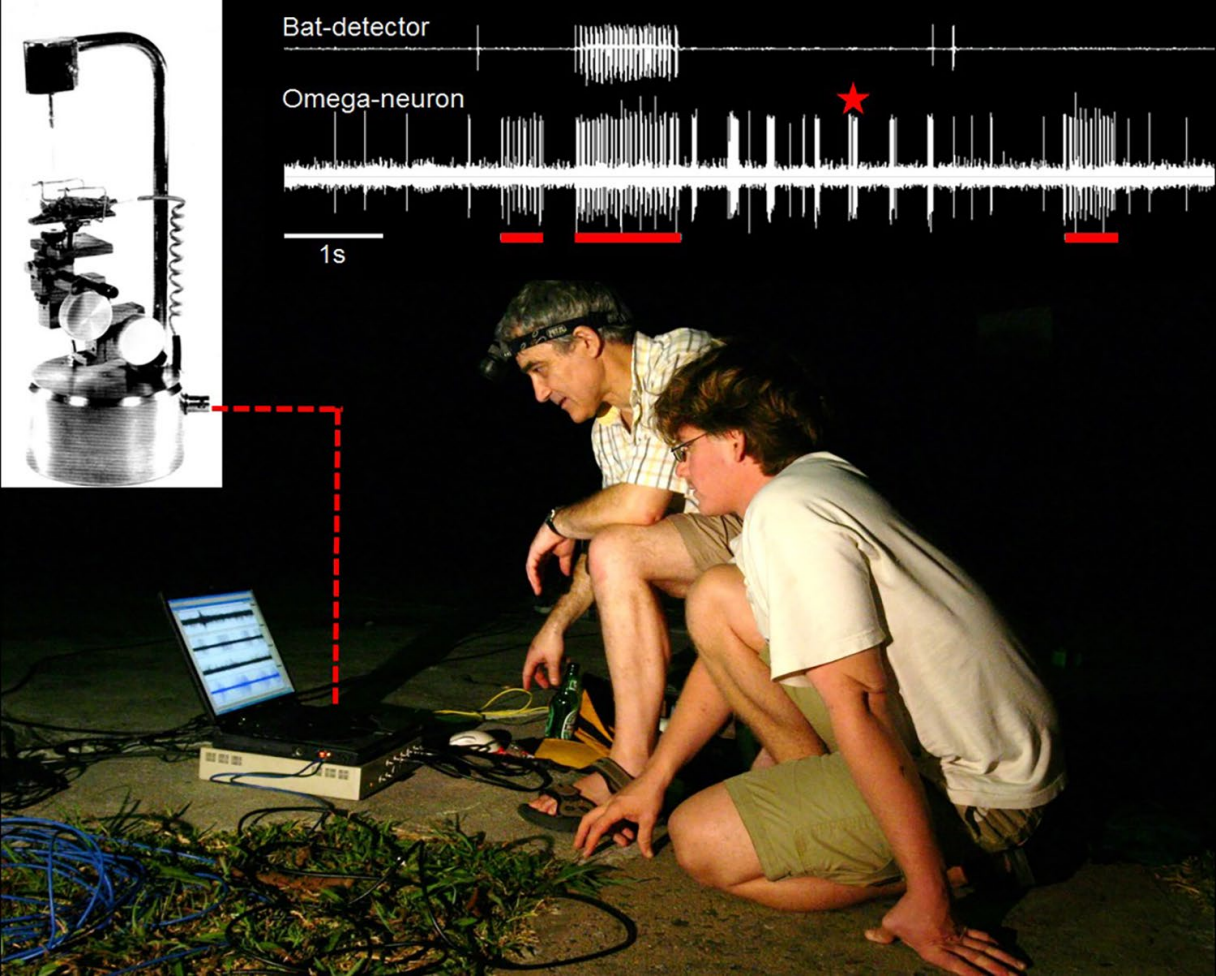
The coding of acoustic events in the nocturnal tropical rainforest of Panama is studied by the author and his PhD-student Alexander Lang, using the “biological microphone approach”. The action potential activity of the omega neuron of a rainforest katydid was recorded with a portable recording unit (left) about 1 h after sunset. A bat detector (upper line) was placed next to the preparation, indicating the highly repetitive echolocation calls of a bat passing by. Note that the neuron fires bursts of APs in response to these calls, but also to other bat calls that are not detected by the bat detector (red bars), most likely due to the different directionality of the technical and biological receivers. Bursts of APs are also elicited by unknown sources and in response to a playback of a short conspecific call of the katydid (asterisk) (Lang, Teppner and Römer, unpublished)

There are several reasons why physiologists left the controlled conditions of a soundproof room to study sensory coding in the animal’s natural environment. Researchers who carried out studies on the effects of the transmission channel on bird song used conventional microphones to quantify temporal distortions or frequency filtering of sound signals. However, for insect sound signals which often include high sonic and ultrasonic frequencies, a microphone placed at the same spot in the environment may not pick up the same sound as the animal receiver (see Fig. 1). The frequency selectivity or tuning of an ear is usually different from the frequency characteristic of a microphone. In a similar way as the A-weighting of a sound level meter with the tuning of a human ear eliminates some of the lower and higher frequencies that are actually present in the sound field, the evolutionary tuning of insect ears frees the CNS from the burden of having to process much of irrelevant sound in the environment (the matched filter hypothesis; Capranica and Moffat 1983; Wehner 1987; for one example, see below). Other differences between technical receivers and insect ears concern their absolute sensitivity, the temporal integration time, and directionality.

Rheinlaender and Römer (1986) called their outdoor recording set-up a “biological microphone” (Fig. 1), because the recordings of AP activity of identified thoracic auditory neurons allowed them to listen to an acoustic scene through the ears of an insect. The first-order omega-neuron is a local neuron in the prothoracic ganglion and thus its activity is not directly forwarded to the brain. However, it integrates sensory information from almost all receptor cells, so that its tuning and sensitivity are almost identical to those of the ear. Furthermore, the AP response of the neuron follows the temporal pattern of an acoustic stimulus, and it receives contralateral inhibition from the mirror-image omega neuron. Altogether, these attributes make outdoor recordings of the activity of the omega cell very suitable for studying sound perception and localization in the field.

Take the recording shown in Fig. 1 (length about 10 s), which was obtained about 1 h after sunset in the tropical rainforest of Panama. The omega neuron of a katydid fires bursts of APs, and very rarely single APs, in response to unknown acoustic events. Because the researchers had placed a bat detector next to the preparation, we can be sure that the high repetition of bursts (~ 20 Hz) was due to the echolocation call of a bat passing by. This situation is quite similar to that Roeder experienced with the moth preparation. The CNS of the insect, like any nervous system, has to interpret what has happened in the outside world from such afferent spike trains. The situation is even more complicated: the single burst of APs marked by an asterisk in Fig. 1 is the response to the short conspecific signal delivered through a speaker. But how does the CNS of the insect discriminate this burst of APs from the many others that appear in the recording as a result of background noise? Redundant signalling would be one solution to decrease the uncertainty in burst identification, but many katydids in the tropical rainforest in Panama produce short duration calls (< 40 ms) at an exceptionally low rate (< 10 s of sound per individual per night; Symes et al. 2016), so that repetitive sampling appears impossible. It is currently completely unknown how males and females of these species, which live in low densities in the rainforest, find each other through phonotaxis with such low duty cycles.

How reliable is the representation of acoustic stimuli in bursts of auditory neurons under these conditions? To this end, Pfeiffer et al. (2012) explored an unsupervised machine learning algorithm based on probabilistic inference to find frequently occurring burst patterns in the responses of the omega neuron, which were recorded under the same conditions as shown in Fig. 1. This allowed to ask how much information the CNS of the receiver can extract from bursts without being told by an assumed “supervisor” which type and which variants of bursts are characteristic for particular stimuli. The results showed that the reliability of burst coding in the time domain was so high that patterns of APs in response to identical stimuli exhibited a high degree of similarity, even for different preparations of the omega neuron recorded on different nights. Future behavioural experiments are badly needed to examine whether females show a reliable phonotactic response under these conditions indicating that their CNS can also discriminate among these different bursts of APs as well as an unsupervised machine learning algorithm.

As the recording in the nocturnal rainforest in Fig. 1 indicates, strong competition within and between species for the airborne sound channel can increase the background noise level, so that the signal-to-noise ratio (SNR) for communication signals decreases and signal detection and/or discrimination is severely impaired (Brumm 2014). Three ways have been reported for insects how the auditory system can reduce the effects of masking noise (Schmidt and Römer 2011; Römer 2014).

If the relevant signal is centred around a given carrier frequency, as in the calling song of crickets, one sensory adaptation would be to narrow the tuning of the ear around the species-specific calling frequency. Thus, any sounds that fall outside the sensitivity range of the filter will play a reduced role in masking the signals, depending on the sharpness of the tuning. Schmidt et al. (2011) studied the frequency tuning of an auditory neuron (AN1 neuron) in the rainforest cricket *Paroecanthus podagrosus* and two species of European field crickets*. P. podagrosus* suffers from strong song competition for the sound channel, whereas such competition does not exist among the European field crickets. As predicted, AN1 in the rainforest species exhibited a more selective tuning as compared to the European counterparts. One important point: the higher selectivity of the filter is mainly due to a steeper slope of the V-shaped tuning curve towards higher frequencies, where the carrier frequencies of several other cricket species occur and compete for the sound channel. How does this affect the sensory representation of a cricket’s calling song in the field under the nocturnal noise conditions?

**Fig. 2:**
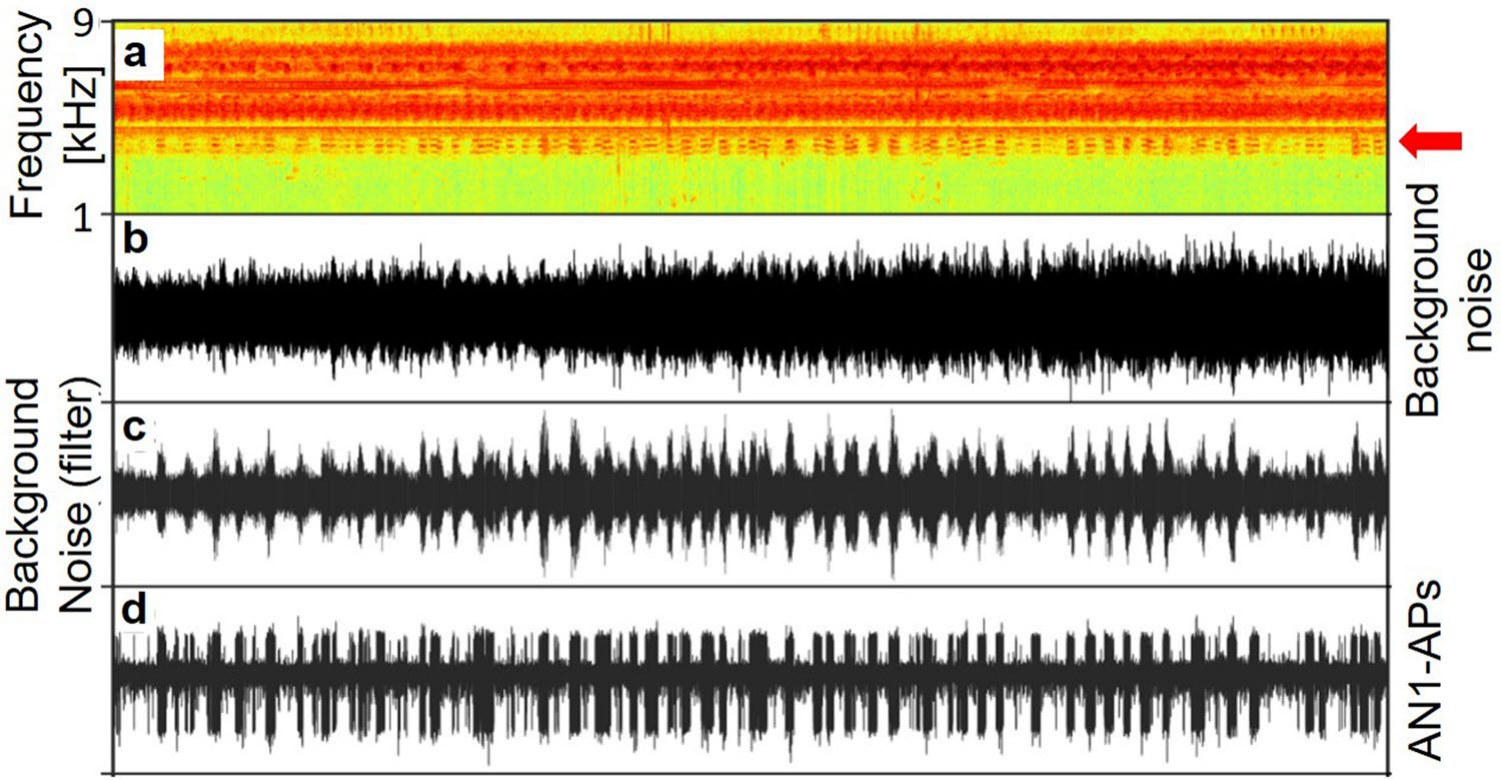
Representative section of 30 s of nocturnal background noise recording as sonogram and oscillogram, respectively (**a**, **b**). Filtering of this sound section with a filter function derived from the tuning curve of AN1 in *Diatrypa* sp*.* (**c**) reveals an amplitude modulation coinciding with the bursting activity of the AN1 neuron (**d**). Bursts were elicited by sound events in the narrow frequency band between 3.5 and 4.5 kHz, representing calling songs of several *Diatrypa* males at various distances from the preparation (arrow in **a**). Modified from Schmidt et al. (2011)

Schmidt and Römer (2011) used the “biological microphone” approach and placed a preparation with an omega-cell recording of a rainforest cricket (*Diatrypa* sp.) with a similar selective tuning outdoors, at a time when conspecific males as well as several different cricket species were calling. This was a test of sensory coding of naturalistic stimuli, i.e. the detection of calling songs of several conspecific males transmitted over unknown distances and embedded in the acoustic background of other acoustic insects. Figure 2 shows a representative section of 30 s of nocturnal background noise presented as a sonogram and oscillogram, respectively (a, b), where the latter shows almost no amplitude modulation. However, when this sound section was filtered with a filter function derived from the tuning curve of AN1 in *Diatrypa* sp*.* (c), an amplitude modulation was revealed which coincides quite nicely with the bursting activity of the neuron (d). These bursts were elicited by sound events in a 1-kHz frequency band between 3.5 and 4.5 kHz, representing calling songs of several *Diatrypa* males at various distances from the preparation (arrow in a). This reflects the excellent performance of the AN1 filter in reducing background noise, especially towards the higher frequencies at which other cricket species were singing.

In addition to the selective tuning, two further ‘bottom-up’ mechanisms contribute to the excellent neuronal representation of conspecific signals despite the strong masking background. Laboratory experiments yielded an average SNR of − 8 dB when the masker and signal were broadcast from the same side. Displacing the masker by 180 degrees from the signal in the laboratory (a common procedure in such experiments from insects to humans) improved the SNRs by another 6–9 dB, a phenomenon known as spatial release from masking (see also Brunnhofer et al. (2016) with values for two other insect species). Surprisingly, when the same preparation with the recording of AN1 activity was tested in the lab and directly in the nocturnal rainforest, SNRs of about − 23 dB were measured in the latter situation, as compared to values of about − 15 dB in the laboratory (Schmidt and Römer 2011).

The significant differences between the laboratory and outdoor results result from the way such masking experiments are usually performed in the lab, whereby the ear faces directly towards a single speaker that broadcasts the masker, which is then shifted to contralateral. Apparently, such single speaker playbacks do not properly reconstruct the noise situation in a spatially realistic manner, because multiple sound sources are spatially distributed (in all three dimensions) in the natural habitat. Thus, under natural conditions where the masking noise acts on the receiver from all directions, the SNR in the masked condition is almost identical to the unmasked threshold in the lab (Schmidt and Römer 2011). This indicates that the effect of masking noise under natural conditions is strongly reduced due to spatial unmasking, provided by both the directionality of the ear and central nervous lateral inhibition.

The third mechanism that contributes to the high performance of signal representation in the auditory system of insects is based on a specific membrane property of nerve cells, such as the omega neuron or AN1. A gain-control mechanism favours only the most intense of several alternative signals in the nervous response (Pollack 1988 for crickets; Römer and Krusch 2000 for katydids). The underlying mechanism is a calcium-driven hyperpolarisation with a slow build-up and decay time (Sobel and Tank 1994; Baden and Hedwig 2006), and the inhibition prevents suprathreshold depolarisation of the membrane in response to softer signals or background noise. The adaptive function of the gain-control mechanism for the sensory coding and the behaviour of receivers under field conditions is obvious: when several signallers are within earshot of a female, this mechanism limits the perception to only the one or two closest males, prevents the confusion of the amplitude pattern, and frees the CNS from the burden of processing irrelevant (more distant) signals. Indeed, in a field study with *Tettigonia viridissima*, almost all females moved from their release sites toward the closest singing males (Arak et al. 1990).

In retrospect, the reason why this phenomenon was discovered relatively late in katydids in our lab was that we chose the “true” natural signal incorrectly: in *T. viridissima*, the song of the male consists of a double syllable element repeated at a high rate for many minutes. Without current computer technology available, some effort was necessary to reconstruct the amplitude modulation of the double syllable with the correct spectral composition, (Dörrscheidt and Rheinlaender 1980), but responses of interneurons were tested with this single double syllable, broadcast once/s, and not with the long series as in the natural song. Because the gain-control mechanism has a long time constant of about five seconds, it could not be elicited with a single double syllable. The lesson from these examples: even when investigating the coding in the sensory periphery, it is important to use the naturally repetitive, long-lasting stimuli correctly, because the way adaptation changes the onset response curve of auditory receptors determines which and how much information about a given stimulus is available at more central stages (Hildebrand et al. 2014 for review).

## Sensory coding of sound direction in the field

Laboratory studies have shown that insects use small IIDs and ITDs in the order of 1 dB and 0.5–1 ms, respectively, as binaural cues for directional hearing, and display a localisation performance similar to that of mammals. Laser Doppler Vibrometry has enabled researchers to measure the minute deflections of tympanal membranes, and sophisticated trackball systems have allowed them to unravel the solutions in various insect for sound localization (Robert 2005; Schöneich and Hedwig 2010; Windmill and Jackson 2016; Römer 2020). However, binaural hearing evolved under the complex acoustic conditions in the field, where the measurement of minute interaural differences is almost impossible. The “biological microphone” approach is quite useful in this context, because ITDs or IIDs do not exist after stimulus transduction. Instead, directionality is represented in the sensory system as binaural discharge differences or time-of-arrival differences, which can be measured using simultaneous, binaural recordings either of auditory receptors in both ears or in pairs of directionally sensitive interneurons in grasshoppers, crickets, and katydids even under field conditions (Gilbert and Elsner 2000; Rheinlaender and Römer 1986; Kostarakos and Römer 2010). By measuring their activity, researchers can obtain a more naturalistic view of the sensory coding of sound direction. One general result of these studies is that directional hearing is not only a property of the biophysical solutions in the various types of ears, but also depends strongly on properties of the transmission channel and the spatial positions of signallers and receivers.

**Fig. 3:**
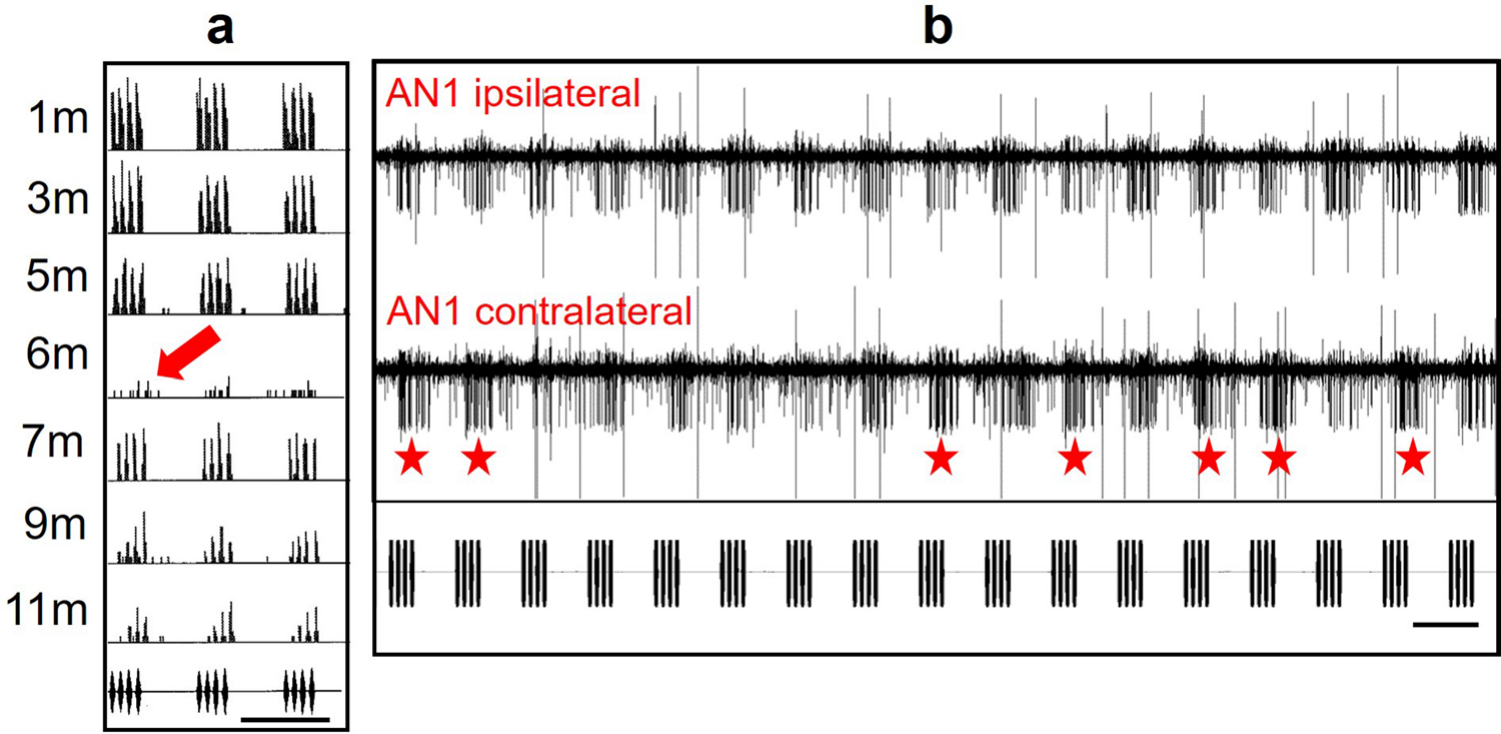
**a** Peri-stimulus time histograms of AN1 activity in *Gryllus bimaculatus* in response to a model of the calling song broadcast at a rate of 3/s at various distances from the source in natural grassland typical for a field cricket. Note the “silent spot” at a distance of 6 m (arrow) with a response at threshold, but with substantial suprathreshold response at larger distances (from Zorn-Pauly and Römer, unpublished). **b** Simultaneous field recording of left and right AN1 activity (smaller APs; larger APs are from AN2 neuron) at a distance of 10 m from the sound source outdoors. The conspecific chirp (lower panel) was presented at a stimulus angle 30° of the longitudinal body axis for the ipsilateral AN1. Note the change of correct and incorrect directional information (contralateral AN1 with stronger activity, asterisks) over time at the same location. In addition to binaural discharge differences, binaural latency differences as potential cues for directional information were analysed. In most experiments, the latency differences closely correlated with the maxima and minima of discharge differences, but at some distances, latency differences were large, whereas the discharge differences approached zero. Time bar in A and B 350 ms. (Kostarakos and Römer unpublished)

For example, although the transmission channel differed strongly in the cricket and katydid studies, under natural conditions positions in the field were identified where the animal could detect the signal, but the directional information in the discharge of the interneurons was completely lost (Rheinlaender and Römer 1986; Kostarakos and Römer 2010). This could have happened at any position along the transect in the cricket study. Even at the same location, the magnitude of directional cues—measured as the discharge difference in bilateral AN1 responses—could vary widely over time, probably as a result of fluctuations in local temperature or wind gradients (Fig. 3b). These aspects add to the irregularities found in the cricket study, i.e. that “silent spots” occurred at various positions within the hearing range, whereby the studied neuron was below threshold (Fig. 3a). The reasons for the loss of directionality in the cricket study are not clear, but the dense vegetation around the katydid might have caused multiple scattering effects that can result in a more or less diffuse sound field, whereby sound waves arrive at the ear from many different directions. This is particularly true for many katydid sound signals in the high audio and ultrasonic ranges, with a wavelength that has the same dimension as the size of scattering vegetation. In addition to a diffuse sound field, dense vegetation in the habitat acts as a frequency-dependent filter (Michelsen 1978; Keuper et al. 1989; Römer and Lewald 1992), and because directional hearing in katydids is strongly frequency dependent (Rheinlaender and Römer 1980; Shen 1993; Schul 1997), the high frequencies providing high directionality may not be available at the positions of the receiver.

Degradation of directional cues can happen over rather short distances. Gilbert and Elsner (2000) compared recordings of directional profiles for auditory receptors of the grasshopper *Ch. biguttulus* in three different types of vegetation with a profile taken in a free sound field. The maximal IID of 24.5 dB available in the free sound field was reduced to 6.8 dB in dense vegetation over a distance of only 1 m.

To better understand the problems associated with directional hearing in the natural environment, it is not enough to simply have knowledge of the degradation of directional cues. As noted above, background noise can mask the signal. If we consider an insect with a high rate of signalling, such as a field cricket with 2–3 chirps/s, the loss of 50% of signals due to masking interference may still allow the receiver to use the remaining, somewhat distorted directional information to perform phonotaxis toward the signaller. Other insects, however, exhibit extremely low signalling rates (Symes et al. 2016). When redundant signalling occurs, the unreliable directional responses of afferent neurons could be sampled over time, an option that is not available for species that signal with low redundancy. For crickets with their redundant signalling two outdoor studies have quantified phonotaxis and demonstrated that all females finally arrived at the target. However, larger deviations were observed in their phonotactic paths when compared with laboratory trials (Mhatre and Balakrishnan 2007; Hirtenlehner and Römer 2014). Future experiments should also consider the possibility that acoustic orientation could be based on a sequential comparison of the acoustic input, when binaural hearing is impaired, as has been suggested earlier for one-eared crickets (Schildberger and Kleindienst 1989), and experimental evidence provided for the moth Achroia grisella (Greenfield et al. 2002; Reid et al. 2016). Reichert (2015) performed a behavioural study with male grasshoppers on the effect of masking noise on their sound localisation abilities. Depending on the level of masking, noise sharply reduced the responsiveness of the males to female songs, as expected. However, in those cases males had detected the signal within noise, they responded highly accurately, even at the highest noise levels. Thus noise strongly affected signal detection, but directional hearing was only weakly impaired.

## A more naturalistic view for sensory coding of bat predator cues

The interaction between bats and their insect prey is one of the best-studied predator–prey relationships. It is also a success story regarding a neuroethological approach to animal behaviour (Hoy et al. 1989; Hoy 1992; Fullard 1998; Miller and Surlykke 2001; Yager 2012; ter Hofstede and Ratcliffe 2016). As noted above, Roeder pioneered the studies in the early 1960s and provided a description of receptor activity in some moths in response to bat echolocation calls (Roeder and Treat 1957). Numerous subsequent reports by James Fullard and collaborators documented the predator–prey relationship between bats and moths, providing strong evidence that their ears evolved as a result of selection pressure applied by echolocating bats (review in ter Hofstede and Ratcliffe 2016). For example, moth ears are typically tuned to the frequencies of the echolocation calls of their sympatric bat community (Fullard 1988, 1998), and the receptors have physiological properties that allow them to maintain sensitivity to the pulsed calls of bats (Fullard et al. 2008). Other taxonomic groups of insects display similar avoidance behaviour to bat calls as moths (Hoy et al. 1989; Fullard and Yack 1993; Yack and Dawson 2008; ter Hofstede and Ratcliffe 2016). In flying crickets the activity in a single auditory interneuron is necessary and sufficient to induce them to steer away from ultrasonic sound pulses (Nolen and Hoy 1984; Hoy et al. 1989). Thus, it appears that in the context of sensory coding of an important predator cue the natural stimulus sufficiently explains the coding and decision strategies used by the receiver’s nervous system.

However, consider the finding of categorical frequency perception in crickets by Wyttenbach et al. (1996). The authors demonstrated that these insects, when on the wing, perform positive phonotaxis towards stimuli below 15 kHz and fly away from sounds with high sonic and ultrasonic frequencies. Such simple labelling of ‘good’ and ‘bad’ frequencies in the decision heuristic creates a significant problem when crickets listen to bat echolocation calls in their natural environment, under conditions of high background noise. In nocturnal rainforests, the noise includes high sonic and ultrasonic frequencies produced by other insects (mainly katydids; Ellinger and Hödl 2003; Lang et al. 2005; Symes et al. 2018), and also the echolocation calls of frugivorous bats in the bat community (Kalko et al. 1996) which do not represent a threat to flying insects. According to the signal detection theory, responding to these calls with bat avoidance behaviour would represent a false alarm (Wiley 2013) and should be avoided. Thus, the insect faces no simple task when trying to navigate the naturalistic acoustic scene and respond both quickly and correctly to a potentially deadly predator.

Small swordtail crickets that live under such conditions are perfectly adapted to cope with the acoustic cues of echolocating bats embedded in the background noise, having found an ideal behavioural solution (Römer and Holderied 2020). Their bat avoidance behaviour exhibits high thresholds of about 80 dB SPL, which is markedly higher than that of most other studied eared insects. At suprathreshold amplitudes, the response is always a short cessation of flight. An analysis of bat and katydid sound amplitudes and peak frequencies in the crickets’ rainforest habitat revealed that the high behavioural threshold would successfully reject the irrelevant katydid background noise. At the same time, the criterion also ignores the low-amplitude bat calls below 80 dB SPL indicating bats which are further away. By measuring the crickets’ echo target strength for bat predators, together with the transmission of bat calls to the target and the echo back to the bat, the detection distances for both predators and prey could be calculated. Despite their high behavioural threshold, the cricket prey still has a significant detection advantage at frequencies of 20–40 kHz. Thus, a simple decision criterion based on a high-amplitude behavioural threshold can be adaptive under the high background noise levels in nocturnal rainforests, enabling the insects to avoid making false alarm responses towards bats that are too far away to pose a risk.

But let us consider for a moment the possibility that the crickets had no such simple threshold mechanism, and instead that they had to discriminate bat calls from irrelevant high-frequency events in the background with their auditory system. They would be unlikely to succeed. Stimuli from different sources may be difficult to discriminate due to their similar physical properties or due to the way the sensory system processes them (Green and Swets 1966; Wiley 2006). Both apply in the context of sensory coding of high-frequency stimuli in crickets. The bat calls and the katydid calls in the background both have a pulsed structure and high-frequency spectra (Symes et al. 2016). The representation of high-frequency sound signals forwarded to the brain events for the brain is provided by a single interneuron (either called AN2 or Int-2) in all cricket species thus far studied (Pollack 2015). If this neuron fires high-frequency bursts of APs (about 200 spikes/s), it initiates escape responses during flight in field crickets (Nolen and Hoy 1984). Marsat and Pollack (2006) have shown that bursts in AN2 code the occurrence of salient peaks in high-frequency stimulus amplitude and predict behavioural escape responses with high reliability.

**Fig. 4:**
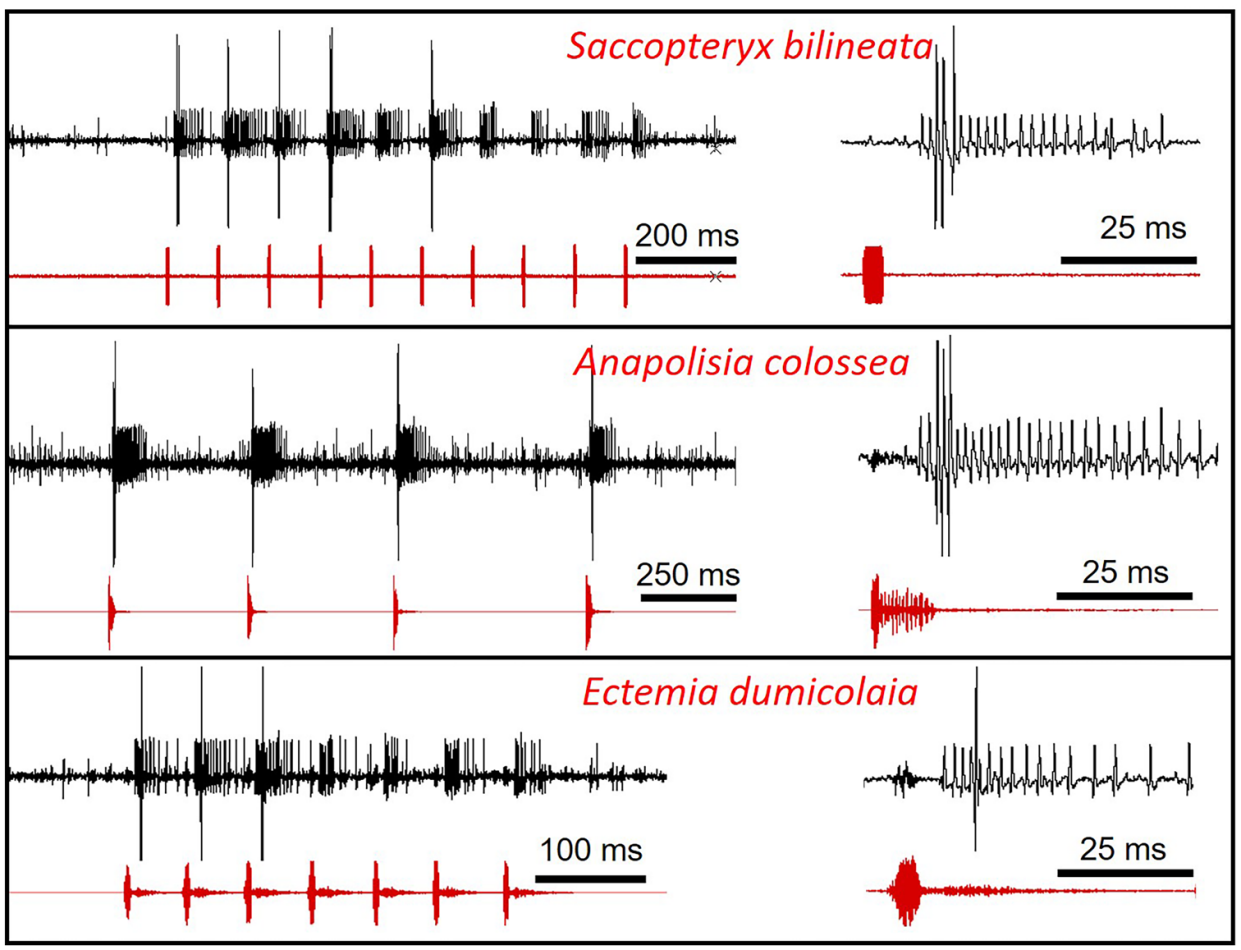
Responses of the likely homologue of AN2 in a rainforest cricket (Int-2; APs with smaller amplitude) to the bat echolocation call of *Saccopteryx bilineata* and to calls of two rainforest katydids (*Anapolisia colossea* and *Ectemia dumicolaia*). Note the similar responses with very high rates of APs to single, short sound pulses (Brunnhofer and Römer, unpublished)

However, an analysis of a suspected homologue of AN2 in a rainforest cricket reveals that it fires strong bursts of action potentials in response to bat echolocation pulses, but also to the short sound pulses in various katydid calls (Fig. 4) with a firing rate up to 700 spikes/s. This is much higher than rates reported for a field cricket (Nolen and Hoy 1984). As crickets have a very limited repertoire of neuronal elements coding for ultrasonic frequencies, it seems unlikely that they could discriminate between the predator cue and high-frequency background events. Given this limitation, therefore, the threshold criterion in the small rainforest crickets is highly adaptive in the natural environment.

For Neotropical katydids that live in habitats with many frugivorous, non-dangerous bat species the presence of echolocation correlates only weakly with the risk of attack. Symes et al. (2020) assessed whether katydids stop calling when exposed to echolocation. Although the insects could detect the predator cues, many species continued calling. Instead, the animals rely on proactive defences (short, infrequent calls lasting less than two cumulative seconds of sound per night). The authors also doubt that katydids can distinguish between the calls of frugivorous and eavesdropping gleaner bats, given the fact that the echolocation calls of most of these species cannot be distinguished on the basis of frequency, duration, or other parameters (Kalko et al. 1996) and due to the limitations of the katydid’s auditory system (Stumpner and Novotny 2014). In the case of wax moths, however, it is the difference in the temporal structure of conspecific calls (ultrasonic clicks delivered at a rate of 80–100/s) and bat calls (short pulses at rates of < 30/s) that can be used for discrimination, despite their similar spectra (Greenfield and Weber 2000). For a more general treatment on how stimulus ambiguity shapes animal decisions, see Leavell and Bernal (2019).

## Conclusion and outlook

As we have seen, insects operate under rather complicated ecological conditions when listening and communicating by sound. Several factors contribute to the fact that stimuli produced by signallers or predators are not those processed by the sensory system of receivers. One factor is the physical structure of the sound transmission channel, which may degrade the temporal pattern and attenuate the signal in unpredictable ways. A second factor is that acoustic communication rarely happens in dyadic interactions between one signaller and receiver, but in choruses of conspecific and heterospecific individuals. This results in reduced signal-to-noise-ratios and imposes challenges for the sensory representation of biologically important stimuli. Barlow’s “efficient coding hypothesis” states that sensory systems are adapted for coding these biologically important stimuli. However, studies have also shown that biases can be established in the nervous system of receivers in a context other than selection of the stimulus trait under study. For this reason, it is often difficult to discriminate between a specific adaptation of a sensory coding property to a stimulus feature and such a receiver bias. Future studies will be able to unravel the “true” adaptations for sensory coding by combining the phylogeny of investigated species with the power of a comparative approach taken with species listening and communicating in different acoustic environments,

To improve our understanding of sound localisation performance of insects, researchers must make biophysical measurements in the laboratory to reveal the establishment of IIDs and ITDs as binaural cues for directional hearing. However, the coding of sound direction is also strongly impaired in the natural environment. Although the investigator faces significant challenges when studying sound localisation in nature as compared to in arena trials or with trackball systems in the lab, more of these behavioural studies must be carried out outdoors to understand whether and how small binaural cues can be used by an insect to approach a target. To this end, we badly need to perform behavioural phonotaxis experiments outdoors with insects using low redundant signalling in the future. In this way, it may be possible to see if and how insects solve the problem of strongly reduced and rare directional information. The “biological microphone” approach can then be used, at least for some model species within the Orthoptera, to complement our view of the “efficient coding” of sound direction as binaural discharge differences. Clearly, we will only be able to complete our understanding of fitness-relevant behaviours under natural conditions by applying an integrative approach, conducting outdoors studies that provide a detailed characterisation of the physical and social environment of acoustic communication in insects and combining these with studying the sensory framework and auditory networks in the brain of receivers for processing the signals.

## Abbreviations

AP: Action potential
CNS: Central nervous system
IID: Interaural intensity difference
ITD: Interaural time difference
SNR: Signal-to-noise ratio
SPL: Sound pressure level
